# Hyperphosphorylation of Tau Associates With Changes in Its Function Beyond Microtubule Stability

**DOI:** 10.3389/fncel.2018.00338

**Published:** 2018-10-09

**Authors:** Alejandra D. Alonso, Leah S. Cohen, Christopher Corbo, Viktoriya Morozova, Abdeslem ElIdrissi, Greg Phillips, Frida E. Kleiman

**Affiliations:** ^1^Department of Biology and Center for Developmental Neuroscience, College of Staten Island, The City University of New York, Staten Island, NY, United States; ^2^Biology Program, The Graduate Center, The City University of New York, New York, NY, United States; ^3^Biochemistry Program, The Graduate Center, The City University of New York, New York, NY, United States; ^4^Department of Biology, Wagner College, Staten Island, NY, United States; ^5^Department of Chemistry, Hunter College, The City University of New York, New York, NY, United States

**Keywords:** tau, PH-tau, hyperphosphorylation, propagation, microtubules, neurodegeneration

## Abstract

Tau is a neuronal microtubule associated protein whose main biological functions are to promote microtubule self-assembly by tubulin and to stabilize those already formed. Tau also plays an important role as an axonal microtubule protein. Tau is an amazing protein that plays a key role in cognitive processes, however, deposits of abnormal forms of tau are associated with several neurodegenerative diseases, including Alzheimer disease (AD), the most prevalent, and Chronic Traumatic Encephalopathy (CTE) and Traumatic Brain Injury (TBI), the most recently associated to abnormal tau. Tau post-translational modifications (PTMs) are responsible for its gain of toxic function. Alonso et al. (1996) were the first to show that the pathological tau isolated from AD brains has prion-like properties and can transfer its toxic function to the normal molecule. Furthermore, we reported that the pathological changes are associated with tau phosphorylation at Ser199 and 262 and Thr212 and 231. This pathological version of tau induces subcellular mislocalization in cultured cells and neurons, and translocates into the nucleus or accumulated in the perinuclear region of cells. We have generated a transgenic mouse model that expresses pathological human tau (PH-Tau) in neurons at two different concentrations (4% and 14% of the total endogenous tau). In this model, PH-Tau causes cognitive decline by at least two different mechanisms: one that involves the cytoskeleton with axonal disruption (at high concentration), and another in which the apparent neuronal morphology is not grossly affected, but the synaptic terminals are altered (at lower concentration). We will discuss the putative involvement of tau in proteostasis under these conditions. Understanding tau’s biological activity on and off the microtubules will help shed light to the mechanism of neurodegeneration and of normal neuronal function.

## Introduction

Tauopathies are a group of dementias that have in common the formation of intracellular filamentous deposits seeded by the microtubule-associated protein tau, in abnormally hyperphosphorylated form(s). These disorders share a common disease mechanism and includes Alzheimer disease (AD), fronto-temporal dementia with Parkinsonism linked to chromosome 17 (FTDP-17), amyotrophic lateral sclerosis, cortical basal degeneration, dementia pugilistica, Pick’s disease, progressive supranuclear palsy, tangle-only dementia, Chronic Traumatic Encephalopathy (CTE) and Traumatic Brain Injury (TBI). Tau inclusions are common among all of these tauopathies leading to diverse phenotypic manifestations, brain dysfunction, and degeneration. These diseases all implicate abnormal tau, with the absence of other disease-specific abnormalities (except in AD), in the onset and/or progression of disease.

Microtubule assembly is promoted and stabilized by the predominantly neuronal protein tau (Weingarten et al., 1975). In the central nervous system, tau has six isoforms derived from a single gene by alternative pro-mRNA splicing (Goedert et al., 1989; Himmler et al., 1989). In the human brain, tau isoforms range in size from 352 to 441 amino acids, with differences in the number of tubulin-binding domain repeats (R), three or four consisting of 31 or 32 amino acids near the C-terminus, or two, one, or no inserts of 29 amino acids near the N-terminus. Under normal conditions, tau is a phosphoprotein, in which isoform expression and degree of phosphorylation are developmentally regulated. However, in the disease state, tau has been found to be abnormally hyperphosphorylated and contains significantly higher phosphate content than the normal tau resulting from the phosphorylation at new sites on the protein (Kopke et al., 1993).

Axonal transport is essential to the growth and survival of a neuron throughout its life. Disruption of microtubules, as are observed in patients with AD, interrupts axonal transport which prevents vesicles and organelles from reaching the synapses. These result in the slow and steady deterioration of the synapses and retrograde degeneration. Mutations of *MAPT*, the tau gene, were discovered in 1998 and co-segregate with the disease in FTDP-17, providing unequivocal evidence that abnormalities in tau alone are enough to cause neurodegenerative disease (Hutton et al., 1998; Poorkaj et al., 1998; Spillantini et al., 1998). We have shown that hyperphosphorylation of tau can result in inhibition of microtubule assembly and can disrupt the preassembled microtubules *in vitro* (Alonso et al., 1994, 1997, 2001b). Though the majority of evidence thus far supports the relationship of tau toxicity with disruption of the microtubule system, emerging evidence suggest that tau biological functions are not restricted to the cytoskeletal system: tau is present in the nuclei (Brady et al., 1995; Greenwood and Johnson, 1995; Frost et al., 2014; Multhaup et al., 2015; Bukar Maina et al., 2016) though this physiological function remains elusive; tau self-acetylation activity has been described but physiological consequences of this modification remains uncertain (Cohen et al., 2013); tau has also been shown to be present in the dendrites under pathological conditions and impair synaptic function (Hoover et al., 2010). More recently, the idea of tau transmitting the disease as a prion-like protein is becoming more attractive. As tau is found in the extracellular space and can be uptaken by neighbor neurons, this tau-mediated pathway might represent an attractive model of therapeutical target to halt neurodegeneration. In this review article, we will discuss the putative mechanisms of tau-induced neurodegeneration in light of the current knowledge and our own experience with tau models.

## Tau: Pathological Gain of Function

### Tau Isoforms and Post-translational Modifications

The gene for tau, *MAPT*, is located on chromosome 17q21.1. It is a single copy gene which undergoes alternative splicing to generate six isoforms found in the human brain (as reviewed in Andreadis, 2005). The number of N-terminal inserts can vary (0N, 1N, or 2N) as well as the number of C-terminal repeats (3R or 4R). These repeats are located in the microtubule binding domain (MTBD), which can lead to differential polymerization rates when mixed with tubulin. When analyzed in microtubule polymerization reactions, recombinant proteins representing each of these six tau isoforms and the 3R proteins had slower rates of polymerization than 4R proteins independently of the N-terminal composition (Goedert and Jakes, 1990). The 4R and 3R isoforms of tau can be found in an approximately 1:1 ratio in normal adult brains with the 1N form at the highest level (50%), 0N (40%) and 2N (10%; Higuchi et al., 2002). Each of the six isoforms have been found in the brains of AD and other tauopathies (including Downs Syndrome, amyotrophic lateral sclerosis, Niemann-Pick disease Type C and some FTDP-17 mutations) at similar ratios as normal brains (Higuchi et al., 2002; Connell et al., 2005). Other tauopathies, including cortical basil degeneration, progressive supranuclear palsy, and other FTDP-17 mutations, appear to express more 4R proteins than 3R proteins at both the mRNA and protein levels (Higuchi et al., 2002; Connell et al., 2005). Brains from Pick’s Disease patients show higher 3R levels than 4R in the sarkosyl insoluble fractions, and this change in the ratio appears to occur post-transcriptionally (Higuchi et al., 2002; Connell et al., 2005).

Tau function is modulated by many post-translational modifications (PTMs) including, but not limited to, phosphorylation, acetylation, ubiquitination and protein fragmentation (as reviewed in Beharry et al., 2014; Alonso et al., 2016). As the review of all of tau PTMs is extremely challenging due to the large number of modified sites and by the coexistence of multiple types of modifications, this review article will focus on how tau changes due to phosphorylation can affect microtubule stability and regulate not fully elucidated tau functions. Many of the PTMs lead to changes in the interaction of tau with other molecules by changing the charge of an amino acid, or by removing a section of the protein as occurs in fragmentation. The cleavage of tau by calpains and/or caspases can result in molecules that are aggregate-prone which can cause other full-length tau molecules to aggregate with it (Chung et al., 2001; Gamblin et al., 2003; Guillozet-Bongaarts et al., 2005; Mondragón-Rodríguez et al., 2008a,b).

### Microtubules and Tau in Alzheimer’s Disease

**Figure 1:**
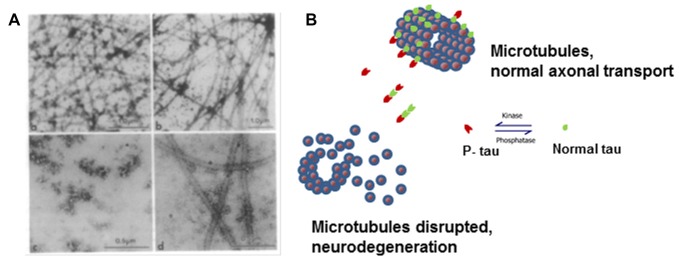
Microtubule disruption can be caused by hyperphosphorylation of tau. **(A)** Electron micrographs showing the products of microtubule assembly. The microtubules were negatively stained with phosphotungstic acid. Rat brain tubulin was placed in an *in vitro* reaction in the presence of **(a)** control acid-soluble tau, **(b)** Alzheimer disease (AD) acid-soluble tau, **(c)** AD Phosphorylated tau (AD P-tau) and **(d)** AD P-tau after dephosphorylation. Very few microtubules were observed in the presence of AD P-tau and dephosphorylation appears to slightly rescue microtubule disruption (Alonso et al., 1994). **(B)** Cartoon model of microtubule disruption due to P-tau sequestration of normal tau away from the tubulin resulting in instability and loss of cytoskeletal structure. Image reproduced as authorized by the editors of Alonso et al. (2016).

In neurons from patients with AD, there is a decrease in microtubules and a several-fold increase in the concentration of tau (Kopke et al., 1993). There are three different pools of tau in the brains of AD patients: AD tau is most similar to normal tau and is not hyperphosphorylated; AD Phosphorylated tau (AD P-tau) is soluble hyperphosphorylated tau; and paired helical filaments (PHFs)-tau is insoluble and hyperphosphorylated. AD tau in AD brains is decreased by about 60% compared to tau found in normal brain. AD P-tau, as well as normally phosphorylated tau, can be isolated from AD brain in solution (Kopke et al., 1993). Analyzing microtubule-promoting activity of tau from AD brains, we found that AD tau has normal activity in *in vitro* assembly of microtubules assays. Conversely, AD P-tau did not promote microtubule assembly but this activity was recovered upon dephosphorylation with alkaline phosphatase treatment (Alonso et al., 1994; Figure 1). Interestingly, we found that AD P-tau inhibited the microtubule assembly promoted by normal tau, MAP1 and MAP2 (Alonso et al., 1997). Pre-incubation of AD P-tau with normal tau prior to the addition to tubulin inhibited not only the normal tau-microtubule–promoting activity but also destroyed microtubules already present. This was probably due to interactions between tau and AD P-tau resulting in the sequestering of normal tau from the tubulin, and suggesting that AD P-tau has prion-like activity.

### AD P-Tau Has Prion-Like Properties

The ability of AD P-tau to bind normal tau was verified using a solid phase binding assay (Alonso et al., 1996). Quantitation of this binding was performed using an *in vitro* assay in solution and we observed that the binding of AD P-tau to normal tau was non-saturable (Alonso et al., 1996). Furthermore, analysis by electron microscopy indicated that the products of these reactions were bundles of filaments (Alonso et al., 1996). Based on these results, we hypothesized that the hyperphosphorylation of tau changed its conformation in such a way that this change could be transferred to the normal protein, acting as a prion-like molecule to seed pathological tau self-assembly. The ability of hyperphosphorylated tau to bind normal tau was also observed in yeast, a less complex cellular model (Vandebroek et al., 2005). Expression in yeast of the human four-repeat and three-repeat isoforms demonstrated that these isoforms became phosphorylated at pathological sites and assumed a pathological conformation resulting in aggregate formation. The phosphorylation was modulated by yeast kinases Mds1 and Pho85, orthologs of GSK-3β and cdk5. The Van Leuven group observed many biochemical characteristics similar to those we had already observed with human tau including a positive correlation between tau aggregation and phosphorylation; slower mobility in SDS-PAGE with increased phosphorylation; increase in the formation of filaments with isolated hyperphosphorylated tau, and induction of nucleation, or seeding, of normal-non-P-tau assembly by this hyperphosphorylated tau (Vandebroek et al., 2005). The prion-like properties of hyperphosphorylated tau are due to its biochemical stability, which promotes the aggregation of tau, and are consistent with our observations using hyperphosphorylated tau isolated from Alzheimer brain (Alonso et al., 1996). The conformational change transfer by AD P-tau to normal tau is similar to a property of a prion protein. We were the first to link hyperphosphorylation of tau to these nucleation properties of tau (Alonso et al., 1994, 1997, 2001a).

### Hyperphosphorylation Induces Prion-Like Tau Self-Assembly

In AD progression, tau becomes hyperphosphorylated prior to the appearance of neurofibrillary tangles (Bancher et al., 1989). Hyperphosphorylated tau shows a 2-3-fold increase in the number of moles of phosphate per mole of protein (Kopke et al., 1993) which results from the appearance of new phosphorylated sites. The increase in phosphorylation can lead to neuronal degeneration triggered by tau self-assembly into tangles composed of PHFs and straight filaments (SFs). In *in vitro* assays at varying pHs, AD P-tau self-assembled into tangles of PHFs mixed with SFs (Alonso et al., 2001b). The PHFs generated were ~20 nm wide which narrowed to ~10 nm at approximately every 80 nm similar to those of AD PHFs. Within the PHFs, protofilaments (4 nm) and SFs (~15 nm) were observed, also similar to those found in AD. Dephosphorylation of AD P-tau inhibited the self-assembly of tau (Alonso et al., 1994), suggesting that hyperphosphorylation was required for filament formation.

Tau intermolecular association leading to self-assembly appears to occur through the MTBD, whereas the flanking regions can inhibit these interactions (von Bergen et al., 2000; Pérez et al., 2001; Alonso et al., 2004). These tau regions have concentrated positively charged residues, and we postulate that these patches might be responsible for the inhibition of tau self-assembly. Supporting this idea, when two N-terminal inserts of tau, which are highly negative, are present, tau self-assembly is induced (Alonso et al., 2001b). Phosphorylation of tau, which introduces negative charges in these regions, results in tau molecules that acquire the ability to bind normal tau. Tau-tau interactions begin to form when there are ~4 moles of phosphate per mole of protein, polymerization of tau leading to fibril formation begins when there are ~10 moles of phosphate per mole of protein (Alonso et al., 2001b, 2004). From these results, we understand that there are at least two different conformational states of tau induced by differential phosphorylation. Similar results are observed by the oxidation of tau by carbonyl addition to Lys residues, which changes positively charged residues to negatively charged ones under oxidative-stress conditions (Santa-María et al., 2005). Consistent with this model, tau molecules modified by truncation, which eliminates the positive fragment, can also induce tau self-assembly (Zhou et al., 2018). In this context, when polyanions are used to induce aggregation, such as RNA, polyGlu, or heparin, it would not be surprising that certain phosphorylation sites appeared to be inhibitory of tau aggregation (Schneider et al., 1999).

To further understand these structural characteristics of tau, six isoforms of tau were expressed heterologously, phosphorylated by treatment with normal brain extract which contains the kinases and we followed their ability to bind normal tau and to inhibit microtubule-promoting activity (Alonso et al., 2001a,b). Proteins treated with rat brain extract became hyperphosphorylated, ~12 moles phosphate per mole of protein (P-tau), which is similar in phospho-level to AD P-tau. These hyperphosphorylated recombinant proteins were able to self-assemble into tangles of PHFs/SFs (Alonso et al., 2001a,b) and inhibit microtubule assembly activity (Alonso et al., 2001b). Interestingly, the presence of FTDP-17 mutations on tau also induced conformational changes (Jicha et al., 1999), decreased the formation of microtubules (Bunker et al., 2006), and/or increased the ability of tau to self-assemble (Goedert et al., 1999). To analyze why these mutations may alter tau activity in a similar manner as phosphorylation, we generated recombinant tau proteins containing FTDP-17 mutations (R406W, P301L, V337M, or G272V) and incubated in brain extract as above (Alonso et al., 2004). The results showed that the presence of these mutations increased the rate and extent of phosphorylation (~16–18 moles of phosphate per mole of protein), suggesting that the mutations induced the conformational changes described above (Alonso et al., 2004). These results indicate that the mutated tau molecules are better substrates for kinases than wild-type tau. This effect was observed by another group that reported higher phosphorylation at Ser202 in FTDP-17 mutant tau (Han et al., 2009). Furthermore, these tau mutants showed filament at lower mole amounts of phosphate per mole of protein (Alonso et al., 2004).

Together, our studies indicate that hyperphosphorylation confers upon tau a toxic property, due to its ability to bind normal tau and MAPs. However, we have previously shown that tau filaments do not inhibit microtubule assembly and do not bind other MAPs potentially due to the neutralization of the tau peptide involved in tau self-assembly (Ganguly et al., 2015) by intermolecular interactions (Alonso et al., 2006). Consistent with this, morphometric study of brain biopsy specimen shows that the decrease in microtubule density in AD patients was unrelated to PHF accumulation (Cash et al., 2003). In aged tau transgenic mice from Takashima’s group, synapse loss was found in the same brain region as hyperphosphorylated tau (Kimura et al., 2007). Based on these findings, we hypothesize that once tau becomes hyperphosphorylated it can bind normal MAPs and disrupt microtubules, resulting in the interruption of axoplasmic transport and in synaptic degeneration. However, upon tau self-assembly, there will be no contact region for normal MAPs thereby no disruption of the microtubule network and axonal transport. This model is in agreement with the results of the European Tau meeting held last year that has determined that in tauopathies, tau in aggregates is always hyperphosphorylated (Mudher et al., 2017).

### What Is Hyperphosphorylated Tau? How Can We Study the Gain of Toxic Function?

**Figure 2:**
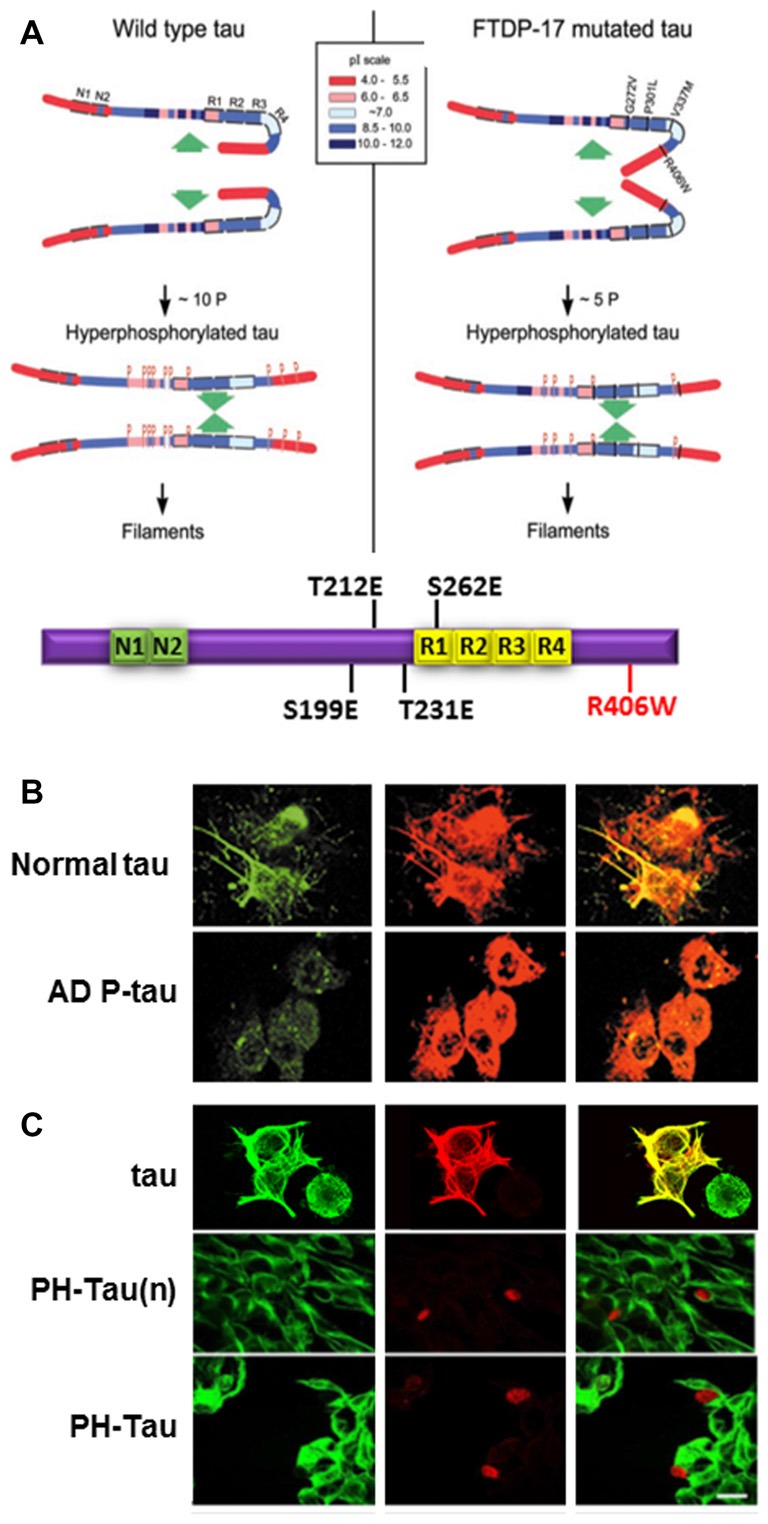
Pathological human tau (PH-Tau) mimics AD P-tau. **(A)** A hypothetical scheme of the phosphorylation-induced self-assembly of wild-type (left) and fronto-temporal dementia with Parkinsonism linked to chromosome 17 (FTDP-17) mutated tau proteins (right). Tau self-assembles mainly through the microtubule binding domain (MTBD)/repeat R2 and R3. The β-structure of R2 and R3 is depicted at the cartoon on the bottom. Regions of tau molecule both N-terminal and C-terminal to the repeats are inhibitory of self-assembly. Hyperphosphorylation of tau neutralizes these basic inhibitory domains, enabling tau-tau interaction. The C-terminal region beyond Pro397 (398–441) is a highly acidic segment masks the repeats. Phosphorylation of tau at Ser396 and/or Ser404 opens this segment, allowing tau-tau interaction through the repeats. FTDP-17 mutations make tau a more favorable substrate for phosphorylation than the wild-type tau. The mutated tau proteins achieve the conformation required to self-assemble at a lower level of incorporated phosphate. Although the FTDP-17 mutant tau proteins have conformations that are more prone to polymerize, in the absence of hyperphosphorylation, the highly basic segments and the C-terminus interfere with polymerization. Phosphorylation sites are indicated by *red P*s at Ser/Thr positions in tau (left panel): 199, 202, 205, 212, 231, 235, 262, 396, 404 and 422; and in FTDP-17 mutant tau (right panel): 199, 212, 231, 262 and 396, respectively. Image taken from Alonso et al. (2004). The Tau cartoon at the bottom indicates the pseudophosphorylated version of tau that is used in our studies, PH-Tau (Di et al., 2016). **(B)** AD P-tau inhibits regeneration of microtubules. To induce microtubule assembly 3T3 cells expressing AD P-tau were incubated with 15% fresh rat brain cytosol in buffer containing 1 mM GTP for 1 h at 37°C. Cells were processed for immunofluorescence staining by using DM1-A Ab against tubulin (green) and 134d rabbit polyclonal Ab against tau (red). AD P-tau, but not normal tau, inhibited microtubule assembly (Alonso et al., 2006). **(C)** PH-Tau, as AD P-tau, inhibits microtubule assembly when expressed in cells. CHO cells were transfected with either tau, PH-Tau on the normal tau background (PH-Tau(n)), or PH-Tau on the R406W background. After 48 h, the cells were permeabilized with 0.1% Nonidet P-40 before fixing and then processed for immunocytochemistry as described in **(B)**. Merge is shown in yellow. Bar, 25 μm (Alonso et al., 2010). This research was originally published by Alonso et al. (2010).

Though the defect in tau that leads to aggregation and neurodegeneration is commonly referred to as hyperphosphorylated tau, its definition is still unclear. The debate is whether tau toxic effect is due to a general increase in moles of phosphate per mole of protein regardless as to where these modifications are or whether there is a need for phosphorylation at specific sites within a molecule. Long-range interaction in tau, and other intrinsically disordered proteins (IDPs), are modulated by intra- and inter-molecular interactions and by PTMs, such as phosphorylation, acetylation and others (Bibow et al., 2011). As an IDP, the secondary structure of tau is not defined, however, tau structural information is very important in the formation of PHF/SF in AD brains (Fitzpatrick et al., 2017). This indicates that our observations on tau self-assembly using the whole molecule hold more weight than those using different fragments of tau. The regions N-terminal to the MTBD are very basic with a pI of more than nine and are separated from other parts of the protein by Pro residues which are known to introduce kinks in protein structure (Figure 2A). The positive charges in this region can mask the negative charges in the MTBD to limit intermolecular interactions. At the start of tau polymerization, three residues in this basic region (Thr212, Thr231 and Ser262) have been shown to be at least 50% phosphorylated, decreasing the pI of the region (Alonso et al., 2004). On the C-terminal side of the MTBD, there is a second basic region and phosphorylation of Ser396 and/or Ser404 may also change the pI, increasing the potential for intermolecular interactions leading to tau self-assembly.

Cells treated with AD P-tau showed decreased microtubule stability (Alonso et al., 2006; Figure 2B). Based on our knowledge of phosphorylation events in AD brains, we used phosphomimetics, with and without the FTDP-17 mutation R406W, to determine whether hyperphosphorylation was site-specific. Residues that were determined to have about five moles of phosphate incorporated per mole of protein when self-assembly occurred were changed to Glu to mimic the negative charge of phosphorylation or to Ala as a non-phosphorylatable control. During the initial analysis, nine sites were found to fit the criteria described above Ser199, Ser202, Ser205, Thr212, Thr231, Ser235, Ser262, Ser396 and Ser404. Vectors were generated by site-directed mutagenesis and then transfected into mammalian cells. Tau proteins containing the Ala mutations expressed in cell lines acted similarly to wild-type tau. Conversely, for most of the Glu mutations some tau dissociation from tubulin was observed, but none exhibited complete microtubule disruption (Alonso et al., 2010). Therefore, single phosphorylation events did not appear to change the charge of the molecule enough to convert tau into an AD P-tau like toxic molecule. To try to mimic the toxic nature of tau, double and triple mutant proteins were expressed in cells, and the triple combination of T212E/S235E/S262E was observed to bind weakly to microtubules with a concomitant decrease in tubulin staining. Furthermore, the triple mutant protein aggregated in the cytoplasm and nuclear space and sequestered normal tau similarly to AD P-tau *in vitro* (Alonso et al., 2010; Figure 2C compared to Figure 2B). A fourth residue, Ser199, was found to be highly phosphorylated in the pseudophosphorylated tau compared to wild-type, suggesting that phosphorylation at this fourth site was able to convert tau into a molecule that had gained toxic function. This toxicity was enhanced by the FTDP-17 mutation R406W (Figure 2C). For our future work, we have focused on a molecule called Pathological Human tau (PH-Tau) containing the phosphomimetics of tau at these four sites (S199, T212, T231 and S262) with the R406W mutation.

### Models Used to Study the Role of Tau in AD and Other Neurodegenerative Disorders

Human tau has been overexpressed in many mouse models which have been shown to reproduce the cognitive impairment found in AD (Berger et al., 2007; Eckermann et al., 2007; Lasagna-Reeves et al., 2011; Roberson et al., 2011; Sydow et al., 2011; Webster et al., 2014) and neuronal death^1^. The level of heterologous tau expression in many of these models is very high compared to levels of endogenous tau which could lead to mechanistic differences in the analysis of the neurons. Furthermore, the analysis gets more complicated since tau has six isoforms, many of these models express variations of human tau, with or without mouse tau, and with or without FTDP-17 mutations (Lewis et al., 2000, 2001; Tanemura et al., 2001, 2002; Tatebayashi et al., 2002; Santacruz et al., 2005; Schindowski et al., 2006; Yoshiyama et al., 2007; Hundelt et al., 2011; Flunkert et al., 2013). Neurodegeneration is observed in most of these models, but these mice also have phenotypes not commonly found in AD, i.e., motor deficiencies due to tau accumulation in the spinal cord (Lewis et al., 2000; Yoshiyama et al., 2007). Many groups have switched to the TetOff system to better regulate tau expression levels and localization. This system utilizes the Calmodulin Cam kinase II promoter which leads to expression in the neurons and can be regulated using doxycycline in the diet (Tatebayashi et al., 2002; Hundelt et al., 2011). Tau expressed in most of these model systems become phosphorylated at common Ser/Thr residues that can be linked to pathogenic phosphorylation sites found in AD as compiled by the Hanger group^2^.

**Figure 3:**
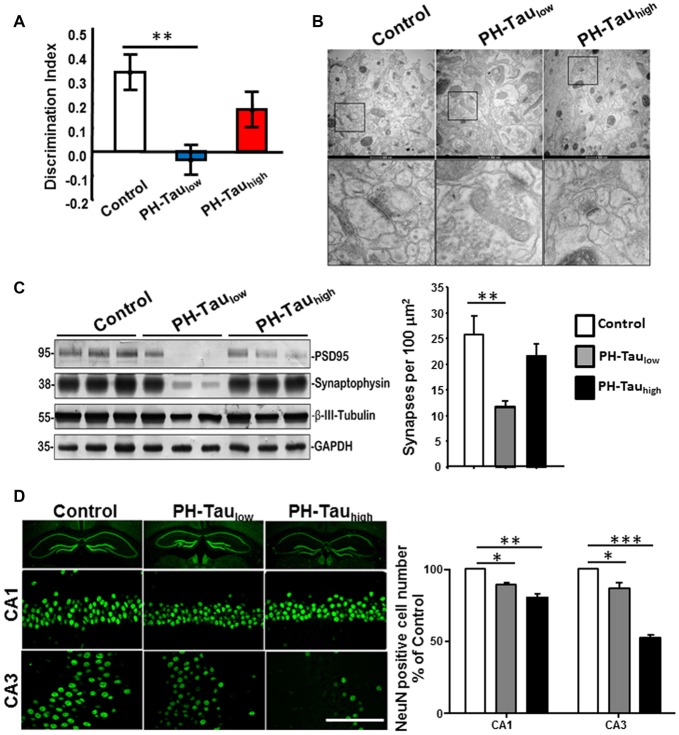
Characterization of PH-Tau mouse model. **(A)** Bigenic mice (12-month old) were tested for behavior deficits in the Novel Object Recognition task. Significant decreases in spatial memory and memory storage were observed. **(B)** PH-Tau_low_ mice shows significant loss of synapses, decreased post-synaptic density and enlarged pre-synaptic portion. Decrease in the length of the post-synaptic density was observed in both PH-Tau_low_ and PH-Tau_high_ mice. Synapses in CA1 stratum radiatum area are shown in the magnified images of square boxes. Quantitation of the number of synapses in the CA1 stratum radiatum area is shown in the bottom of the figure. **(C)** Representative Western blot of mouse hippocampus homogenate. The levels of synaptophysin, PSD95 and β-III-tubulin were measured. Loss of synaptic proteins was observed in PH-Tau_low_ mice. **(D)** Coronal slices of hippocampus stained with antibody NeuN recognizing nucleus of neurons (Left). Scale bar = 50 μm. Counts of the NeuN positive cells in both the CA1 and CA3 regions. *p < 0.05, **p < 0.01, ***p < 0.001; right; Di et al. (2016).

A top priority in the research of neurodegenerative disease is the development of therapeutics. The mouse models that are generated to express tau phosphomimetics may be a useful tool in developing and analyzing new treatments for dementia related to the hyperphosphorylation of tau. However, as described above, one must be careful to use pseudophosphorylation patterns that induce similar conformational changes in tau to those observed in AD P-tau. For example, a mouse model of hyperphosphorylation which studied a tau molecule containing 10 different pseudophosphorylated residues was generated with no cognitive impairment or neurodegeneration observed (Hundelt et al., 2011). Conversely, our studies, including cell and neuronal culture, *Drosophila*, and a mouse model, which target four specific sites (S199, T212, T231 and S262) on tau resulted in cell death in culture and neurodegeneration and learning deficiencies in *Drosophila* and mice (Alonso et al., 2010; Beharry et al., 2013; Di et al., 2016). Furthermore, we showed that the toxic effect was stronger when the sites chosen for pseudophosphorylation were paired with the FTDP-17 mutation R406W (Alonso et al., 2010; Beharry et al., 2013). In our mouse model that uses the TetOff system, we found that PH-Tau was expressed in the forebrain of the mouse and resulted in the formation of aggregates, synaptic disruption, neurodegeneration, astrocytosis and cognitive decline (Di et al., 2016; Figure 3). In this model, PH-tau expression is regulated by the addition (suppressed) or removal (induced) of doxycycline to/from the food and/or water of these animals. When expression was induced, levels of PH-Tau increased up to 14% of total tau protein (i.e., 14 molecules of PH-Tau per 100 molecules of murine tau) and aggregates were detected. When expression was suppressed, there were baseline levels of PH-Tau observed. At this low level, PH-Tau was detected biochemically as oligomers and triggered early cognitive deficits (Figure 3A), and loss of synapses in the hippocampus as determined by decreases in synaptic protein levels and quantitation of electron micrographs (Figures 3B,C). While PH-Tau was barely detectable by immunohistochemistry in tissue sections from these animals, some PH-Tau was observable and appeared to have translocated in the nucleus. Dramatic neuronal loss was not observed in those animals (Figure 3D), but the synaptic changes suggest that at low levels the effect of abnormal tau expression might not be related to changes in the cytoskeleton but to its presence in the nucleus and regulation of protein expression. Interestingly, when PH-Tau expression was induced, cognitive decline was somewhat rescued (Figure 3A), the oligomeric forms of PH-Tau decreased but the sarkosyl-insoluble tau increased, and the decrease of synaptic proteins and synaptic terminals appeared to be reversed (Figures 3B,C). We can speculate that at higher levels, PH-Tau begins to seed a larger amount of self- aggregation as observed by the formation of sarkosyl-insoluble tau and that this may prevent the toxic protein from entering the nucleus. In fact, tau localization was observed in the perinuclear region of the neurons compared to the nuclear localization in the low expressing brains (data not shown). Neuronal death increased in both the CA1 and CA3 regions of the hippocampus from the mice in which PH-Tau expression was induced (Figure 3D). By increasing the expression of PH-Tau, which resulted in the increase of its aggregation propensity into sarkosyl insoluble aggregates, the impairment of cytoskeletal function begins potentially by the sequestering of normal tau from the microtubules, thereby increasing their instability (Di et al., 2016). These results are comparable to the effects of hyperphosphorylated tau in the brains of AD patients. Based on these different observations, it appears that the specific sites that are phosphorylated or modified, rather than the number of sites, may be an important factor in tau toxicity.

## Conclusions and Future Directions

**Figure 4:**
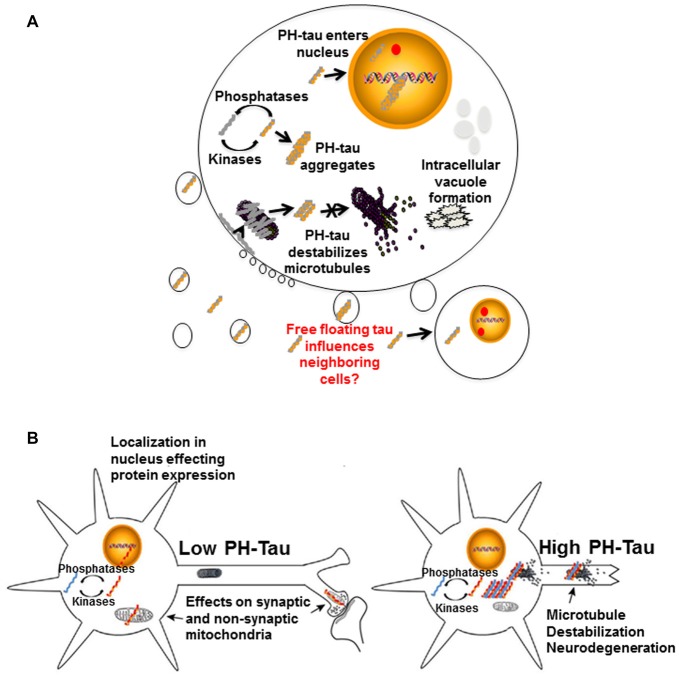
Proposed mechanisms of neurodegeneration. **(A)** Hyperphosphorylation of tau affects multiple cellular processes. PH-tau aggregates are formed when the balance between kinases and phosphorylases is disrupted. The aggregates can begin to disrupt the stability of the microtubules. This toxic molecule has also been shown to translocate into the nucleus, cause intracellular degeneration, protein aggregation and vacuole formation. The presence of tau in the nucleus might be involved in alterations of protein expression. Disruption of the actin cytoskeleton can lead to membrane zeiosis and tau can be released from the cells, potentially propagating the disease to neighboring cells. Image reproduced from Alonso et al. (2016). Permissions were obtained through RightsLink License Number: 4358781157689. **(B)** Left panel: low level of PH-tau expression results in translocation to the nucleus, synaptic dysfunction and mitochondrial disruption. The presence of tau in the nucleus might be involved in alterations of protein expression. Right panel: high levels of PH-Tau expression results in protein aggregation, microtubule disruption and loss of synapses. Loss of cytoskeletal stability leads to neurodegeneration (Alonso et al., 2017). Permission granted to reproduce figure from Alonso et al. (2017).

It is apparent that tau hyperphosphorylation is an early event in the disease progression for AD and other tauopathies, though there are other modifications that can also affect the protein. From all that we have learnt through our research and those of our fellow scientists, we can propose that these modifications of tau can modulate different events at the cellular levels with important consequences for its physiology (Figure 4), including converting the protein from a microtubule stabilizer to a microtubule disrupter inducing a pathological state. Changes in the cytoskeleton might not be the only effect of these mdifications on tau. In our lab, tau has been observed to translocate into the cell nucleus (Alonso et al., 2010; Di et al., 2016). Though tau has been shown to interact with nuclear DNA, hyperphosphorylation can alter this interaction with potential changes at the chromatin and transcriptional level, thereby changing its physiological role (Hua and He, 2003; Wei et al., 2008; Padmaraju et al., 2010; Sultan et al., 2011; Qi et al., 2015). Our preliminary studies suggest that tau in the nucleus may also be involved in the regulation of mRNA stability, which would result in potential changes at the transcriptome and proteome by regulating gene expression, thus affecting cellular function during the progression of neurodegeneration. We have recently found that tau can associate with factors involved in mRNA 3′ processing, such as the tumor suppressor p53 (Devany et al., 2013) and PARN deadenylase (Cevher et al., 2010), and that the formation of tau/p53/PARN complex(es) in the nucleus can regulate mRNA 3′ end processing. Interestingly, these interactions are regulated by tau phosphorylation state (Baquero et al., 2015). These changes might happen at very early stages of disease progression.

We have described above how hyperphosphorylated tau can destabilize the microtubules but it can also interact with actin causing a destabilization of these microfilaments (Elie et al., 2015). Disruption of the microfilaments can lead to zeiosis of the cell membrane due to their important role in membrane stability. We have observed that as membrane pinches off during exocytosis, there is a release of hyperphosphorylated tau within the vesicles. These tau-containing vesicles have the potential to be taken up through endocytosis, or some other mechanism, into neighboring cells. We were the first to show that hyperphosphorylated tau sequesters healthy tau protein causing it to take on the toxic function of the pathological protein (Alonso et al., 1996). These findings support the prion-like nature of hyperphosphorylated tau which can spread its pathology to surrounding cells by moving from cell to cell and sequestering healthy tau which causes a disruption of all cytoskeleton components, destabilization of the organelles, disruption of protein synthesis and eventually zeiosis induction.

Zeiosis of the cell membrane is expected to release hyperphosphorylated tau-containing membrane vesicles. While we do not know the physiological role, if any, of tau in the extracellular space, it is possible that soluble hyperphosphorylated tau is uptaken by non-affected neurons through its interaction with the muscarinic receptor triggering the signal transduction pathway (Gómez-Ramos et al., 2006, 2008). Supporting this model, our preliminary results indicate that the muscarinic receptors are involved in tau’s uptake (Morozova et al., 2017). It is also possible that hyperphosphorylated tau-containing membrane vesicles are taken up by endocytosis by surrounding cells. This model of tau transmission might be therefore addressed with immunotherapy (Iqbal et al., 2018).

Different scenarios can be considered where the levels of hyperphosphorylated tau begin increasing to toxic levels due to kinase overactivity, phosphatase deficiency, failure in the clearance system or a combination of these and others. Early in disease progression, modified tau may translocate into the nucleus, move to synapses, or interfere with mitochondrial homeostasis (Figure 4B, left). Any or all of these physiological changes could lead to cognitive impairment without significant structural changes, as observed in our mouse model under conditions of low PH-Tau expression (Di et al., 2016). As disease progression continues, the levels of pathological tau tend to increase in the neurons to levels that begin to disrupt microtubule stability and other cytoskeletal components which can trigger retrograde neurodegeneration (Figure 4B, right). Despite these different mechanisms, it appears that therapeutics to reduce the levels of hyperphosphorylated tau as well as therapeutics aimed at preventing cytoskeleton disruption remain as key targets for tauopathies (Corbo and Alonso, 2011). The development of combination therapies that address the multiple physiological changes induced by hyperphosphorylated tau may help to unravel the process of neurodegeneration and reduce the number of patients affected.

## Author Contributions

AA is the lead principal investigator (PI) and has contributed in all the experiments and overseen the writing process. LC contributed actively with the writing along with AE (contributor in the behavioral studies), GP (studies of tissue electron microscopy), CC and VM (contributed with cell experiments). All contributed to the writing of the manuscript. FK collaborated with AA in the studies of mRNA processing and tau involvement in the cleavage of the polyA tail. FK wrote the entire manuscript and contributed particularly in the tau-DNA interactions.

## Conflict of Interest Statement

The authors declare that the research was conducted in the absence of any commercial or financial relationships that could be construed as a potential conflict of interest.

## Footnotes

^1^Alzforum.Org

^2^https://docs.google.com/spreadsheets/d/1hGYs1ZcupmTnbB7n6qs1r_WVTXHt1O7NBLyKBN7EOUQ/edit#gid=0

## References

[B1] Alonso A. D. Beharry C. Corbo C. P. Cohen L. S. (2016). Molecular mechanism of prion-like tau-induced neurodegeneration. Alzheimers Dement. 12, 1090–1097. 10.1016/j.jalz.2015.12.014 27126544

[B2] Alonso A. D. Cohen L. S. Morozova V. (2017). “The tau misfolding pathway to dementia,” in Protein Folding Disorders in the Central Nervous System, eds Ghiso J. Rostagno A. (New York, NY: World Scientific Publishing), 83–107.

[B3] Alonso A. D. Di Clerico J. Li B. Corbo C. P. Alaniz M. E. Grundke-Iqbal I.. (2010). Phosphorylation of tau at Thr212, Thr231, and Ser262 combined causes neurodegeneration. J. Biol. Chem. 285, 30851–30860. 10.1074/jbc.M110.110957 20663882 PMC2945578

[B4] Alonso A. D. Grundke-Iqbal I. Iqbal K. (1996). Alzheimer’s disease hyperphosphorylated tau sequesters normal tau into tangles of filaments and disassembles microtubules. Nat. Med. 2, 783–787. 10.1038/nm0796-783 8673924

[B5] Alonso A. D. Grundke-Iqbal I. Barra H. S. Iqbal K. (1997). Abnormal phosphorylation of tau and the mechanism of Alzheimer neurofibrillary degeneration: sequestration of microtubule-associated proteins 1 and 2 and the disassembly of microtubules by the abnormal tau. Proc. Natl. Acad. Sci. U S A 94, 298–303. 10.1073/pnas.94.1.298 8990203 PMC19321

[B6] Alonso A. D. Li B. Grundke-Iqbal I. Iqbal K. (2006). Polymerization of hyperphosphorylated tau into filaments eliminates its inhibitory activity. Proc. Natl. Acad. Sci. U S A 103, 8864–8869. 10.1073/pnas.0603214103 16735465 PMC1482669

[B7] Alonso A. D. Mederlyova A. Novak M. Grundke-Iqbal I. Iqbal K. (2004). Promotion of hyperphosphorylation by frontotemporal dementia tau mutations. J. Biol. Chem. 279, 34873–34881. 10.1074/jbc.M405131200 15190058

[B8] Alonso A. D. Zaidi T. Grundke-Iqbal I. Iqbal K. (1994). Role of abnormally phosphorylated tau in the breakdown of microtubules in Alzheimer disease. Proc. Natl. Acad. Sci. U S A 91, 5562–5566. 10.1073/pnas.91.12.5562 8202528 PMC44036

[B9] Alonso A. D. Zaidi T. Novak M. Barra H. S. Grundke-Iqbal I. Iqbal K. (2001a). Interaction of tau isoforms with Alzheimer’s disease abnormally hyperphosphorylated tau and *in vitro* phosphorylation into the disease-like protein. J. Biol. Chem. 276, 37967–37973. 10.1074/jbc.M105365200 11495914

[B10] Alonso A. D. Zaidi T. Novak M. Grundke-Iqbal I. Iqbal K. (2001b). Hyperphosphorylation induces self-assembly of tau into tangles of paired helical filaments/straight filaments. Proc. Natl. Acad. Sci. U S A 98, 6923–6928. 10.1073/pnas.121119298 11381127 PMC34454

[B12] Andreadis A. (2005). Tau gene alternative splicing: expression patterns, regulation and modulation of function in normal brain and neurodegenerative diseases. Biochim. Biophys. Acta 1739, 91–103. 10.1016/j.bbadis.2004.08.010 15615629

[B13] Bancher C. Brunner C. Lassmann H. Budka H. Jellinger K. Wiche G.. (1989). Accumulation of abnormally phosphorylated tau precedes the formation of neurofibrillary tangles in Alzheimer’s disease. Brain Res. 477, 90–99. 10.1016/0006-8993(89)91396-6 2495152

[B14] Baquero J. Ordonez M. Alonso A. D. Kleinman F. E. (2015). “Tau phosphorylation plays a role in mRNA 3′ end processing,” in Proceedings of the 4th Metabolism in Neurological Disease and 11th Brain Research Conference, Society for Neuroscience, New York, NY.

[B15] Beharry C. Alaniz M. E. Alonso A. D. (2013). Expression of Alzheimer-like pathological human tau induces a behavioral motor and olfactory learning deficit in *Drosophila melanogaster*. J. Alzheimers Dis. 37, 539–550. 10.3233/jad-130617 23948901

[B16] Beharry C. Cohen L. S. Di J. Ibrahim K. Briffa-Mirabella S. Alonso A. D. (2014). Tau-induced neurodegeneration: mechanisms and targets. Neurosci. Bull. 30, 346–358. 10.1007/s12264-013-1414-z 24733656 PMC5562659

[B17] Berger Z. Roder H. Hanna A. Carlson A. Rangachari V. Yue M.. (2007). Accumulation of pathological tau species and memory loss in a conditional model of tauopathy. J. Neurosci. 27, 3650–3662. 10.1523/JNEUROSCI.0587-07.2007 17409229 PMC6672413

[B18] Bibow S. Ozenne V. Biernat J. Blackledge M. Mandelkow E. Zweckstetter M. (2011). Structural impact of proline-directed pseudophosphorylation at AT8, AT100, and PHF1 epitopes on 441-residue tau. J. Am. Chem. Soc. 133, 15842–15845. 10.1021/ja205836j 21910444

[B19] Brady R. M. Zinkowski R. P. Binder L. I. (1995). Presence of tau in isolated nuclei from human brain. Neurobiol. Aging 16, 479–486. 10.1016/0197-4580(95)00023-8 7566354

[B20] Bukar Maina M. Al-Hilaly Y. K. Serpell L. C. (2016). Nuclear tau and its potential role in Alzheimer’s disease. Biomolecules 6:9. 10.3390/biom6010009 26751496 PMC4808803

[B21] Bunker J. M. Kamath K. Wilson L. Jordan M. A. Feinstein S. C. (2006). FTDP-17 mutations compromise the ability of tau to regulate microtubule dynamics in cells. J. Biol. Chem. 281, 11856–11863. 10.1074/jbc.M509420200 16495230

[B22] Cash A. D. Aliev G. Siedlak S. L. Nunomura A. Fujioka H. Zhu X.. (2003). Microtubule reduction in Alzheimer’s disease and aging is independent of tau filament formation. Am. J. Pathol. 162, 1623–1627. 10.1016/S0002-9440(10)64296-4 12707046 PMC1851211

[B23] Cevher M. A. Zhang X. Fernandez S. Kim S. Baquero J. Nilsson P.. (2010). Nuclear deadenylation/polyadenylation factors regulate 3′ processing in response to DNA damage. EMBO J. 29, 1674–1687. 10.1038/emboj.2010.59 20379136 PMC2876964

[B24] Chung C. W. Song Y. H. Kim I. K. Yoon W. J. Ryu B. R. Jo D. G.. (2001). Proapoptotic effects of tau cleavage product generated by caspase-3. Neurobiol. Dis. 8, 162–172. 10.1006/nbdi.2000.0335 11162250

[B25] Cohen T. J. Friedmann D. Hwang A. W. Marmorstein R. Lee V. M. (2013). The microtubule-associated tau protein has intrinsic acetyltransferase activity. Nat. Struct. Mol. Biol. 20, 756–762. 10.1038/nsmb.2555 23624859 PMC3827724

[B26] Connell J. W. Rodriguez-Martin T. Gibb G. M. Kahn N. M. Grierson A. J. Hanger D. P.. (2005). Quantitative analysis of tau isoform transcripts in sporadic tauopathies. Mol. Brain Res. 137, 104–109. 10.1016/j.molbrainres.2005.02.014 15950767

[B27] Corbo C. P. Alonso A. D. (2011). Therapeutic targets in Alzheimer’s disease and related tauopathies. Prog. Mol. Biol. Transl. Sci. 98, 47–83. 10.1016/B978-0-12-385506-0.00002-8 21199770

[B28] Devany E. Zhang X. Park J. Y. Tian B. Kleiman F. E. (2013). Positive and negative feedback loops in the p53 and mRNA 3′ processing pathways. Proc. Natl. Acad. Sci. U S A 110, 3351–3356. 10.1073/pnas.1212533110 23401530 PMC3587245

[B29] Di J. Cohen L. S. Corbo C. P. Phillips G. R. El Idrissi A. Alonso A. D. (2016). Abnormal tau induces cognitive impairment through two different mechanisms: synaptic dysfunction and neuronal loss. Sci. Rep. 6:20833. 10.1038/srep20833 26888634 PMC4757872

[B30] Eckermann K. Mocanu M. M. Khlistunova I. Biernat J. Nissen A. Hofmann A.. (2007). The β-propensity of Tau determines aggregation and synaptic loss in inducible mouse models of tauopathy. J. Biol. Chem. 282, 31755–31765. 10.1074/jbc.M705282200 17716969

[B31] Elie A. Prezel E. Guérin C. Denarier E. Ramirez-Rios S. Serre L.. (2015). Tau co-organizes dynamic microtubule and actin networks. Sci. Rep. 5:9964. 10.1038/srep09964 25944224 PMC4421749

[B32] Fitzpatrick A. W. P. Falcon B. He S. Murzin A. G. Murshudov G. Garringer H. J.. (2017). Cryo-EM structures of tau filaments from Alzheimer’s disease. Nature 547, 185–190. 10.1038/nature23002 28678775 PMC5552202

[B33] Flunkert S. Hierzer M. Löffler T. Rabl R. Neddens J. Duller S.. (2013). Elevated levels of soluble total and hyperphosphorylated tau result in early behavioral deficits and distinct changes in brain pathology in a new tau transgenic mouse model. Neurodegener. Dis. 11, 194–205. 10.1159/000338152 22797329

[B34] Frost B. Hemberg M. Lewis J. Feany M. B. (2014). Tau promotes neurodegeneration through global chromatin relaxation. Nat. Neurosci. 17, 357–366. 10.1038/nn.3639 24464041 PMC4012297

[B35] Gamblin T. C. Chen F. Zambrano A. Abraha A. Lagalwar S. Guillozet A. L.. (2003). Caspase cleavage of tau: linking amyloid and neurofibrillary tangles in Alzheimer’s disease. Proc. Natl. Acad. Sci. U S A 100, 10032–10037. 10.1073/pnas.1630428100 12888622 PMC187753

[B36] Ganguly P. Do T. D. Larini L. LaPointe N. E. Sercel A. J. Shade M. F.. (2015). Tau assembly: the dominant role of PHF6 (VQIVYK) in microtubule binding region repeat R3. J. Phys. Chem. B 119, 4582–4593. 10.1021/acs.jpcb.5b00175 25775228 PMC4428543

[B37] Goedert M. Jakes R. (1990). Expression of separate isoforms of human tau protein: correlation with the tau pattern in brain and effects on tubulin polymerization. EMBO J. 9, 4225–4230. 10.1002/j.1460-2075.1990.tb07870.x 2124967 PMC552204

[B38] Goedert M. Jakes R. Crowther R. A. (1999). Effects of frontotemporal dementia FTDP-17 mutations on heparin-induced assembly of tau filaments. FEBS Lett. 450, 306–311. 10.1016/s0014-5793(99)00508-6 10359094

[B39] Goedert M. Spillantini M. G. Jakes R. Rutherford D. Crowther R. A. (1989). Multiple isoforms of human microtubule-associated protein tau: sequences and localization in neurofibrillary tangles of Alzheimer’s disease. Neuron 3, 519–526. 10.1016/0896-6273(89)90210-9 2484340

[B40] Gómez-Ramos A. Díaz-Hernández M. Cuadros R. Hernández F. Avila J. (2006). Extracellular tau is toxic to neuronal cells. FEBS Lett. 580, 4842–4850. 10.1016/j.febslet.2006.07.078 16914144

[B41] Gómez-Ramos A. Díaz-Hernández M. Rubio A. Miras-Portugal M. T. Avila J. (2008). Extracellular tau promotes intracellular calcium increase through M1 and M3 muscarinic receptors in neuronal cells. Mol. Cell. Neurosci. 37, 673–681. 10.1016/j.mcn.2007.12.010 18272392

[B42] Greenwood J. A. Johnson G. V. (1995). Localization and *in situ* phosphorylation state of nuclear tau. Exp. Cell Res. 220, 332–337. 10.1006/excr.1995.1323 7556441

[B43] Guillozet-Bongaarts A. L. Garcia-Sierra F. Reynolds M. R. Horowitz P. M. Fu Y. Wang T.. (2005). Tau truncation during neurofibrillary tangle evolution in Alzheimer’s disease. Neurobiol. Aging 26, 1015–1022. 10.1016/j.neurobiolaging.2004.09.019 15748781

[B44] Han D. Qureshi H. Y. Lu Y. Paudel H. K. (2009). Familial FTDP-17 missense mutations inhibit microtubule assembly-promoting activity of tau by increasing phosphorylation at Ser202 *in vitro*. J. Biol. Chem. 284, 13422–13433. 10.1074/jbc.M901095200 19304664 PMC2679442

[B45] Higuchi M. Trojanowski J. Lee V. M. (2002). “Tau protein and tauopathy,” in Neuropsychopharmacology: The Fifth Generation of Progress, eds Davis K. L. Charney D. Coyle J. T. Nemeroff C. (Philadelphia, PA: Lippincott, Williams and Wilkins), 1339–1354.

[B46] Himmler A. Drechsel D. Kirschner M. W. Martin D. W. Jr. (1989). Tau consists of a set of proteins with repeated C-terminal microtubule-binding domains and variable N-terminal domains. Mol. Cell. Biol. 9, 1381–1388. 10.1128/mcb.9.4.1381 2498649 PMC362554

[B47] Hoover B. R. Reed M. N. Su J. Penrod R. D. Kotilinek L. A. Grant M. K.. (2010). Tau mislocalization to dendritic spines mediates synaptic dysfunction independently of neurodegeneration. Neuron 68, 1067–1081. 10.1016/j.neuron.2010.11.030 21172610 PMC3026458

[B48] Hua Q. He R. Q. (2003). Tau could protect DNA double helix structure. Biochim. Biophys. Acta 1645, 205–211. 10.1016/s1570-9639(02)00538-1 12573250

[B49] Hundelt M. Fath T. Selle K. Oesterwind K. Jordan J. Schultz C.. (2011). Altered phosphorylation but no neurodegeneration in a mouse model of tau hyperphosphorylation. Neurobiol. Aging 32, 991–1006. 10.1016/j.neurobiolaging.2009.06.007 19660835

[B50] Hutton M. Lendon C. L. Rizzu P. Baker M. Froelich S. Houlden H.. (1998). Association of missense and 5′-splice-site mutations in tau with the inherited dementia FTDP-17. Nature 393, 702–705. 10.1038/31508 9641683

[B51] Iqbal K. Liu F. Gong C. X. (2018). Recent developments with tau-based drug discovery. Expert Opin. Drug Discov. 13, 399–410. 10.1080/17460441.2018.1445084 29493301

[B52] Jicha G. A. Rockwood J. M. Berenfeld B. Hutton M. Davies P. (1999). Altered conformation of recombinant frontotemporal dementia-17 mutant tau proteins. Neurosci. Lett. 260, 153–156. 10.1016/s0304-3940(98)00980-x 10076890

[B53] Kimura T. Yamashita S. Fukuda T. Park J. M. Murayama M. Mizoroki T.. (2007). Hyperphosphorylated tau in parahippocampal cortex impairs place learning in aged mice expressing wild-type human tau. EMBO J. 26, 5143–5152. 10.1038/sj.emboj.7601917 18007595 PMC2140104

[B54] Kopke E. Tung Y. C. Shaikh S. Alonso A. D. Iqbal K. Grundke-Iqbal I. (1993). Microtubule-associated protein tau. Abnormal phosphorylation of a non-paired helical filament pool in Alzheimer disease. J. Biol. Chem. 268, 24374–24384. 8226987

[B55] Lasagna-Reeves C. A. Castillo-Carranza D. L. Sengupta U. Clos A. L. Jackson G. R. Kayed R. (2011). Tau oligomers impair memory and induce synaptic and mitochondrial dysfunction in wild-type mice. Mol. Neurodegener. 6:39. 10.1186/1750-1326-6-39 21645391 PMC3224595

[B56] Lewis J. Dickson D. W. Lin W. L. Chisholm L. Corral A. Jones G.. (2001). Enhanced neurofibrillary degeneration in transgenic mice expressing mutant tau and APP. Science 293, 1487–1491. 10.1126/science.1058189 11520987

[B57] Lewis J. McGowan E. Rockwood J. Melrose H. Nacharaju P. Van Slegtenhorst M.. (2000). Neurofibrillary tangles, amyotrophy and progressive motor disturbance in mice expressing mutant (P301L) tau protein. Nat. Genet. 25, 402–405. 10.1038/78078 10932182

[B58] Mondragón-Rodríguez S. Basurto-Islas G. Santa-Maria I. Mena R. Binder L. I. Avila J.. (2008a). Cleavage and conformational changes of tau protein follow phosphorylation during Alzheimer’s disease. Int. J. Exp. Pathol. 89, 81–90. 10.1111/j.1365-2613.2007.00568.x 18336525 PMC2525766

[B59] Mondragón-Rodríguez S. Mena R. Binder L. I. Smith M. A. Perry G. Garcia-Sierra F. (2008b). Conformational changes and cleavage of tau in Pick bodies parallel the early processing of tau found in Alzheimer pathology. Neuropathol. Appl. Neurobiol. 34, 62–75. 10.1111/j.1365-2990.2007.00853.x 17971079

[B60] Morozova V. Cohen L. S. Alonso A. D. (2017). Receptor Mediated Prion-Like Propagation of PH-Tau. Washington, DC: Society for Neuroscience Meeting.

[B61] Mudher A. Colin M. Dujardin S. Medina M. Dewachter I. Alavi Naini S. M.. (2017). What is the evidence that tau pathology spreads through prion-like propagation? Acta Neuropathol. Commun. 5:99. 10.1186/s40478-017-0488-7 29258615 PMC5735872

[B62] Multhaup G. Huber O. Buée L. Galas M. C. (2015). Amyloid precursor protein (APP) metabolites APP intracellular fragment (AICD), Aβ42, and Tau in nuclear roles. J. Biol. Chem. 290, 23515–23522. 10.1074/jbc.r115.677211 26296890 PMC4583011

[B63] Padmaraju V. Indi S. S. Rao K. S. (2010). New evidences on Tau-DNA interactions and relevance to neurodegeneration. Neurochem. Int. 57, 51–57. 10.1016/j.neuint.2010.04.013 20435075

[B64] Pérez M. Arrasate M. Montejo De Garcini E. Muñoz V. Avila J. (2001). *In vitro* assembly of tau protein: mapping the regions involved in filament formation. Biochemistry 40, 5983–5991. 10.1021/bi002961w 11352733

[B65] Poorkaj P. Bird T. D. Wijsman E. Nemens E. Garruto R. M. Anderson L.. (1998). Tau is a candidate gene for chromosome 17 frontotemporal dementia. Ann. Neurol. 43, 815–825. 10.1002/ana.410430617 9629852

[B66] Qi H. Cantrelle F.-X. Benhelli-Mokrani H. Smet-Nocca C. Buée L. Lippens G.. (2015). Nuclear magnetic resonance spectroscopy characterization of interaction of Tau with DNA and its regulation by phosphorylation. Biochemistry 54, 1525–1533. 10.1021/bi5014613 25623359

[B67] Roberson E. D. Halabisky B. Yoo J. W. Yao J. Chin J. Yan F.. (2011). Amyloid-β/Fyn-induced synaptic, network and cognitive impairments depend on tau levels in multiple mouse models of Alzheimer’s disease. J. Neurosci. 31, 700–711. 10.1523/JNEUROSCI.4152-10.2011 21228179 PMC3325794

[B68] Santacruz K. Lewis J. Spires T. Paulson J. Kotilinek L. Ingelsson M.. (2005). Tau suppression in a neurodegenerative mouse model improves memory function. Science 309, 476–481. 10.1126/science.1113694 16020737 PMC1574647

[B69] Santa-María I. Smith M. A. Perry G. Hernández F. Avila J. Moreno F. J. (2005). Effect of quinones on microtubule polymerization: a link between oxidative stress and cytoskeletal alterations in Alzheimer’s disease. Biochim. Biophys. Acta 1740, 472–480. 10.1016/j.bbadis.2004.11.024 15949717

[B70] Schindowski K. Bretteville A. Leroy K. Bégard S. Brion J. P. Hamdane M.. (2006). Alzheimer’s disease-like tau neuropathology leads to memory deficits and loss of functional synapses in a novel mutated tau transgenic mouse without any motor deficits. Am. J. Pathol. 169, 599–616. 10.2353/ajpath.2006.060002 16877359 PMC1698785

[B71] Schneider A. Biernat J. von Bergen M. Mandelkow E. Mandelkow E. M. (1999). Phosphorylation that detaches tau protein from microtubules (Ser262, Ser214) also protects it against aggregation into Alzheimer paired helical filaments. Biochemistry 38, 3549–3558. 10.1021/bi981874p 10090741

[B72] Spillantini M. G. Murrell J. R. Goedert M. Farlow M. R. Klug A. Ghetti B. (1998). Mutation in the tau gene in familial multiple system tauopathy with presenile dementia. Proc. Natl. Acad. Sci. U S A 95, 7737–7741. 10.1073/pnas.95.13.7737 9636220 PMC22742

[B73] Sultan A. Nesslany F. Violet M. Bégard S. Loyens A. Talahari S.. (2011). Nuclear tau, a key player in neuronal DNA protection. J. Biol. Chem. 286, 4566–4575. 10.1074/jbc.M110.199976 21131359 PMC3039398

[B74] Sydow A. Van der Jeugd A. Zheng F. Ahmed T. Balschun D. Petrova O.. (2011). Tau-induced defects in synaptic plasticity, learning and memory are reversible in transgenic mice after switching off the toxic Tau mutant. J. Neurosci. 31, 2511–2525. 10.1523/JNEUROSCI.5245-10.2011 21325519 PMC6623704

[B75] Tanemura K. Akagi T. Murayama M. Kikuchi N. Murayama O. Hashikawa T.. (2001). Formation of filamentous tau aggregations in transgenic mice expressing V337M human tau. Neurobiol. Dis. 8, 1036–1045. 10.1006/nbdi.2001.0439 11741399

[B76] Tanemura K. Murayama M. Akagi T. Hashikawa T. Tominaga T. Ichikawa M.. (2002). Neurodegeneration with tau accumulation in a transgenic mouse expressing V337M human tau. J. Neurosci. 22, 133–141. 10.1523/JNEUROSCI.22-01-00133.2002 11756496 PMC6757582

[B77] Tatebayashi Y. Miyasaka T. Chui D. H. Akagi T. Mishima K. Iwasaki K.. (2002). Tau filament formation and associative memory deficit in aged mice expressing mutant (R406W) human tau. Proc. Natl. Acad. Sci. U S A 99, 13896–13901. 10.1073/pnas.202205599 12368474 PMC129794

[B78] Vandebroek T. Vanhelmont T. Terwel D. Borghgraef P. Lemaire K. Snauwaert J.. (2005). Identification and isolation of a hyperphosphorylated, conformationally changed intermediate of human protein tau expressed in yeast. Biochemistry 44, 11466–11475. 10.1021/bi0506775 16114883

[B79] von Bergen M. Friedhoff P. Biernat J. Heberle J. Mandelkow E. M. Mandelkow E. (2000). Assembly of tau protein into Alzheimer paired helical filaments depends on a local sequence motif (^306^VQIVYK^311^) forming β structure. Proc. Natl. Acad. Sci. U S A 97, 5129–5134. 10.1073/pnas.97.10.5129 10805776 PMC25793

[B80] Webster S. J. Bachstetter A. D. Nelson P. T. Schmitt F. A. Van Eldik L. J. (2014). Using mice to model Alzheimer’s dementia: an overview of the clinical disease and the preclinical behavioral changes in 10 mouse models. Front. Genet. 5:88. 10.3389/fgene.2014.00088 24795750 PMC4005958

[B81] Wei Y. Qu M. H. Wang X. S. Chen L. Wang D. L. Liu Y.. (2008). Binding to the minor groove of the double-strand, tau protein prevents DNA from damage by peroxidation. PLoS One 3:e2600. 10.1371/journal.pone.0002600 18596978 PMC2432501

[B82] Weingarten M. D. Lockwood A. H. Hwo S. Y. Kirschner M. W. (1975). A protein factor essential for microtubule assembly. Proc. Natl. Acad. Sci. U S A 72, 1858–1862. 10.1073/pnas.72.5.1858 1057175 PMC432646

[B83] Yoshiyama Y. Higuchi M. Zhang B. Huang S. M. Iwata N. Saido T. C.. (2007). Synapse loss and microglial activation precede tangles in a P301S tauopathy mouse model. Neuron 53, 337–351. 10.1016/j.neuron.2007.01.010 17270732

[B84] Zhou Y. Shi J. Chu D. Hu W. Guan Z. Gong C. X.. (2018). Relevance of phosphorylation and truncation of tau to the etiopathogenesis of Alzheimer’s disease. Front. Aging Neurosci. 10:27. 10.3389/fnagi.2018.00027 29472853 PMC5810298

